# Is the Response of Tumours Dependent on the Dietary Input of Some Amino Acids or Ratios among Essential and Non-Essential Amino Acids? All That Glitters Is Not Gold

**DOI:** 10.3390/ijms19113631

**Published:** 2018-11-17

**Authors:** Francesco S. Dioguardi, Vincenzo Flati, Giovanni Corsetti, Evasio Pasini, Claudia Romano

**Affiliations:** 1 Determinants of Metabolism Research Laboratory, 20137 Milan, Italy; 2 Department of Biotechnological and Applied Clinical Sciences, University of L’Aquila, 67100 L’Aquila, Italy; vincenzo.flati@univaq.it; 3 Department of Clinical and Experimental Sciences, University of Brescia, Viale Europa, 11-25124 Brescia, Italy; giovanni.corsetti@unibs.it (G.C.); cla300482@gmail.com (C.R.); 4 Istituti Clinici Scientifici Maugeri IRCCS, Cardiac Rehabilitation of the Institute of Lumezzane (Brescia), 25065 Brescia, Italy; evpasini@gmail.com

**Keywords:** amino acids, cancer, energy metabolism, autophagy, apoptosis, glutamine, diabetes type 2

## Abstract

Energy production is the main task of the cancer cell metabolism because the costs of duplicating are enormous. Although energy is derived in cells by dismantling the carbon-to-carbon bonds of any macronutrient, cancer nutritional needs for energetic purposes have been studied primarily as being dependent on glycolysis. Since the end of the last century, the awareness of the dependence of cancer metabolism on amino acids not only for protein synthesis but also to match energy needs has grown. The roles of specific amino acids such as glutamine, glycine and serine have been explored in different experimental conditions and reviewed. Moreover, epidemiological evidence has revealed that some amino acids used as a supplement for therapeutic reasons, particularly the branched-chain ones, may reduce the incidence of liver cancer and a specific molecular mechanism has been proposed as functional to their protective action. By contrast and puzzling clinicians, the metabolomic signature of some pathologies connected to an increased risk of cancer, such as prolonged hyperinsulinemia in insulin-resistant patients, is identified by elevated plasma levels of the same branched-chain amino acids. Most recently, certain formulations of amino acids, deeply different from the amino acid compositions normally present in foods, have shown the power to master cancer cells epigenetically, slowing growth or driving cancer cells to apoptotic death, while being both beneficial for normal cell function and the animal’s health and lifespan. In this review, we will analyze and try to disentangle some of the many knots dealing with the complexities of amino acid biology and links to cancer metabolism.

## 1. Introduction

Since Otto Warburg described the abundant amounts of energy necessary for duplication, derived in tumours from cytoplasmic glycolysis to lactate, even under aerobic conditions [1], the finalistic advantages of incomplete glycolysis for cancer cells and reduced mitochondrial glycolysis, the so-called “Warburg effect”, are still debated [2,3]. The main task of cancer cells is to duplicate, and this requires both energy and substrates adequate to complete the synthesis of any structure, allowing for the formation and survival of new cells. Therefore, the quality and quantity of substrates present in the tissue metabolic environment drive the fate of normal cells [4] and probably select cancer cells from their beginning [5]. Protein synthesis is the most energy demanding because each peptide bond has a cost of 4 ATP [6], and, among the few advancements in understanding the peculiarities of energy production by cancer metabolism, most are related to the observation that cancer cells use glutamine far beyond the need for protein synthesis [7].

Some of the pathways controlling the matching of energy and substrates for synthesis have been identified, and different kinases have been studied in both normal and cancer cells [8,9,10]. Attempts have been made to identify molecules that control metabolic pathways at key points, so that if blocked they would impair selectively cancer cell development and survival, but most have failed [11]. Until now, few molecules have been proven to be effective in inhibiting cancer development or they have been invariably toxic to normal cells [12,13,14,15].

In the new century, attention to the amino acid and protein synthesis metabolism in cancer cells has grown. Most researchers have focused on the possible role of different non-essential (NE) amino acids (AA) in cancer cell growth because their carbon skeletons may provide key intermediates for energy metabolism. Accordingly, renewed attention to some specific NEAA metabolic pathways has led to the discovery of novel biochemical interactions, such as the allosteric activation by the exogenous serine of pyruvate kinase [16], the enzyme that de-phosphorylates the terminal glycolytic intermediate phospho-enol-pyruvate to pyruvate, and whose activation and significance in cancer remains controversial [17].

Although historic studies have identified the source of amino acids used by cancer in the same tissues that serve as a reservoir during prolonged starvation [18], amino acids that are important for cancer energy metabolism and syntheses include proline, glutamine, glycine and serine, with the latter three being the most abundant amino acids in food proteins. Regarding glycine and serine, which are strictly related metabolically, a very recent report suggests that eliminating them by diet would antagonize tumour development, based on data both in vitro and in vivo [19].

By contrast, a few weeks later, another study reported that two formulations comprising mainly essential AA (EAA), which also provides serine and ornithine-αketoglutarate (αKG), potential precursors of glutamine synthesis, were both efficient in inducing apoptosis in different cancer cell lines. This study showed that EAA in excess of NEAA creates a paraphysiological condition of substrates unlikely available in plasma and, thus to the cancer cell metabolism. This amino acid availability is healthy for normal cells but causes fragility in cancer cells [20], leading to the selection of clones [21] adapted to a substrate providing an environment conditioned by nutrition and a protein composition where NEAA are always prevalent; thus, cancer cells are fully dependent on NEAA in excess for development, survival, and multiplication. The study was planned to explore the hypothesis that the most relevant competitive advantage of cancer cells is based on the prevalent availability of NEAA, which are more abundant in food and body proteins than EAA necessary for synthesis to reduce energy costs because the production of NEAA would consume energy and subtract key glycolytic intermediates necessary to provide the carbon backbone to maintain synthesis. This metabolic behaviour would differently control the activation or inactivation of pathways connected with survival like autophagy or proteolysis, which are used by cancer cells to refuel energy continuously and synthetic paths of substrates necessary for rapid duplication. The balance between energy and synthesis is controlled by the usually far fewer EAA provided by food or protein dismantling are usually scarcely provided by plasma and poorly available for normal or cancer cells. Inversion of the EAA/NEAA ratios unveils the dependence of cancer cells on the abundance of NEAA because, when EAA are sufficiently present to drive synthesis, proteasome-dependent proteolysis is inhibited, but autophagy is triggered to match NEAA needs at ratios adequate to maintain synthesis at the high pace dictated by EAA availability. Thus, EAA abundance markedly outdoes the evidently not compelling reciprocal controls predicted by the actual knowledge of the mammalian target of rapamycin (mTOR) and adenosine monophospate- activated protein kinase (AMPK) relationship and opens a different scenario about understanding inhibition or activation of autophagy. On the other hand, the proteasome inhibition exerted by EAA, different from that exerted by drugs like bortezomib or carfilzomib [22], is particularly evident and limited to cancer cells. Furthermore, EAA have already been shown to be protective against doxorubicin heart damage [23], characterized by the dysregulation of mitochondria and autophagy [24], and were also proven to be efficient in ameliorating heart performance in aged diabetic patients affected by heart failure [25]. The introduction of serine and ornithine-αketoglutarate (OKG), in specific ratios with EAA, seems efficient to further implement the effects of altering the availability of EAA/NEAA, and this not-so-predictable result is particularly efficient in eliciting the activity and control of endoplasmic reticulum proteases, specifically by inducing cathepsin-L, which is mainly involved in triggering autophagy.

Is it possible that two different experimental protocols produced such similar results but different conclusions by the authors? What is the role of serine and glycine and why are they so frequently involved in cancer metabolism? What is the biochemical relationship among the serine-glycine pathway, glutamine and proline and energy metabolism?

## 2. Nutrition and the Risk of Cancer: The Puzzling Question of Insulin Resistance and Type-2 Diabetes. Are Amino Acid Plasma Patterns the Cause or Effect?

The matter is relevant because insulin resistance and hyperinsulinemia, unfortunately, intertwined with type 2 diabetes, are considered risk factors for cancer, and this risk has been linked to the insulin hyper-activation of phosphatidylinositol kinase (PI3K)/α-serine/threonine kinase (AKT)/mTOR Complex 1/mTORC1) signalling [26]. Thus, in a recent report, Fontana et al. highlighted the possible role of increased insulin resistance detected when branched chain amino acids (BCAA) were supplemented in the context of a high-fat diet in rats [27], although they do not further discuss the finding that BCAA have no effect on insulin resistance when added to normal chow, as observed in the same paper they cited [28]. Additionally, another report showed that doubling dietary leucine intake significantly reduced either high-fat-diet-induced weight gain and improved hyperglycaemia and hypercholesterolaemia because insulin resistance was also reduced [29]. We also observed that a double-blind controlled study in humans on long-term therapy with BCAA supplementation in diabetic patients that found positive results regarding glucose tolerance and the typical features of insulin resistance and liver steatosis [30] and another study showing that the acute ingestion of BCAA elicits an efficient insulin response and consequent hypoglycaemia [31] were not considered and discussed. For further clarification, a very well-designed study showed that leucine supplementation in high-fat diets would have different effects on glucose tolerance and insulin resistance if studied in the short or long term when beneficial effects are unequivocally evident. In the first 24 weeks, mitochondria damage by fat loads prevails and overwhelms the positive effects of leucine supplementation observed only after 32 weeks when mitochondria biogenesis and glucose tolerance are restored by leucine [32].

However, contrasting opinions exist concerning the role of essential amino acids in promoting health and lifespan [33]. Although it was proven in animal settings that the supplementation of EAA, rich in BCAA, increases the lifespan by protecting mitochondrial biogenesis and implementing mitochondria efficiency-related systems (i.e., endothelial nitric oxide synthase: eNOs, cytochrome c, reactive oxygen species: ROS, and antioxidant defense expression) [34,35], Fontana et al. claimed that branched-chain amino acid restriction is a possible promoter of health, a hypothesis based also on the preliminary observation that the plasma profiles of diabetes type 2 patients are characterized by high levels of BCAA [27].

An elevation of branched-chain amino acids in the plasma of type 2 diabetes patients is often suspected to play a causal role in diabetes as reported in many studies; thus, by contrast, elevated BCAA in plasma are not considered as a potential biomarker of an altered metabolism following the reduced peripheral catabolism of BCAA. This acritical interpretation of metabolomics is puzzling for experts in amino acid metabolism because, in type 2 diabetes patients, both BCAA transaminase and branched-chain ketoacid dehydrogenase are decreased in adipose tissue, and mitochondrial oxidation is diminished in peripheral muscles [36], and the sum of those modifications explain plasma BCAA elevation in those patients.

Notably, in another pathological condition also characterized by the presence of insulin resistance and altered glucose metabolism, liver cirrhosis, BCAA in plasma are so often reduced that they are related to a specific metabolic alteration of brain function, a syndrome known as hepatic encephalopathy [37]. There is no clear explanation for the diverging plasma patterns of BCAA in those two clinical pictures characterized by insulin resistance, but we may link the differences to metabolic insufficiency and the altered haemodynamics of a damaged liver.

Additionally, it is helpful to clarify the picture of the extremely complex protocol of Fontana et al. [27]. They reduced the nitrogen content of diets to about 7% in mice and compared the consequent physical and biochemical modifications with findings observed in a control group fed with 21% grams of amino acids. Calculated from their tables, amino acids provided by the diets were 7.5 and 24.45 g, respectively. Regarding the percentage of total nitrogen, the diets provided 56–62% of NEAA, whether cysteine and tyrosine are considered functionally as NEAA or EAA. Consequently, BCAA representing 39–44% of all EAA always depend on how cysteine and tyrosine would be classified functionally. Indeed, both amino acids may be considered somehow essential in cell culture because tyrosine is indispensable for all cells except for the liver and partially the kidneys, the only organs containing the hydroxylase of phenyl-alanine providing tyrosine synthesis, while sulphur-containing amino acids are most safely provided by balanced methionine and cysteine-cystine stoichiometric ratios, thus, protecting the folate metabolism and minimizing homocysteine synthesis due to methionine metabolism to match cysteine requirements [20]. Following a reduction of nitrogen intake to one third, an almost 30% increased daily caloric intake per the of body weight when deprived diets were given, they observed a continuous drop in body weight by losses of both fat and lean body mass. These findings could identify an efficient model of “sarcopenia [38] leading to wasting [39]”, but this achievement was not extensively discussed. By contrast, although animals fed control diets had normal glucose tolerance, the authors identified an “improved glucose tolerance” related to a lowered glycaemia and insulinaemia in animals fed the near 7% protein diets and with losses of muscles and adipose tissue compared with the controls. Any effect on glucose and insulin, as well as on muscle and fat wasting, was abolished, and the findings were comparable to those of the controls by a further diet containing 21% amino acids, the same amount provided in control diets. However, the formula was modified and contained enormously increased amounts of some neoglucogenic non-essential (NE) AA (glycine, almost 5 fold; proline and serine, 2.7 fold; alanine, more than 2 fold; aspartic acid, almost 30%) while providing markedly (30% or less) reduced amounts of other NEAA (arginine, cysteine, glutamine, and tyrosine) and all the essential amino acids, with the exception of BCAA, whose content was not modified. Thus, in this latter diet, BCAA were contained in the same amounts provided by the control diets and were in extremely elevated ratios compared with all other essential amino acids. The skilled biochemist would remember both the allosteric inhibition exerted by alanine on both 6-phosphofructokinase and pyruvate-kinase and the competitive relationship between alanine and pyruvate on pyruvate transporters for mitochondrial entry, explaining, although arginine was reduced, why the overwhelmingly increased amounts of neoglucogenic glycine, serine, aspartic and glutamic acid would have blunted any other biological effects of EAA. Consequently, any alteration eventually observed would not be purely attributable to the elevated amounts of BCAA provided by this diet [27]. Although the elevation of insulin and glycaemia were not detected compared with the control diets, the hypothesis of a protective effect of BCAA towards those types of dietary amino acid manipulations was not even considered by the authors.

Concluding remarks derived by those data are that, while there is evidence proving that alimentary amino acids—that is, the sum of EAA and NEAA in the ratios contained in foods—may promote insulin resistance [40], in an argument discussed in details elsewhere [41], Fontana et al. indeed showed by this complicated protocol that a marked increase in BCAA, even under those demanding conditions, would not be associated with rising insulinaemia and glycaemia at levels different from what was observed by the control diets. However, they claimed, even by the title of their paper, that the decreased consumption of BCAA improves metabolic health.

Because we are conducting very straightforward and rigorous protocol lifespan studies dealing with varying EAA/NEAA ratios at 15% steps in diets from 100% NEAA up to 100% EAA, it would have been interesting to have data on the length of the lifespan of animals wasted by the 7% protein diet, and if, at necroscopy, alterations of both weight and histochemistry of internal organs were detected, as well as if those matched with what they defined as “improved glycemic control” compared with insulinaemia and glycaemia registered in normal animals fed normal diets [42]. Indeed, should it be considered an improvement that can be linked to increased lifespan, the reduction of glycaemia and insulinaemia in healthy animals, following diets providing marked nitrogen restriction and signed by the caloric increase, but so deprived of nitrogen to induce both losses of adipose tissue and muscle mass? The question posed by Fontana et al. deals with the definition of normality and is extraordinarily relevant because they suggested a finalistic interpretation of changes in glycaemia and insulinaemia found in wasted animals that must be compared with those we would usually have considered normal because the study was performed in normal animals with normally active beta cells that were fed with normal diets, and not in animals affected by insulin resistance.

It would be interesting to answer this question, and implications are enormous to define and understand the relationship among nitrogen intake, body composition, and lifespan. It would also be important to clarify the metabolic systems controlling the synthesis of fats in adipose tissue and proteins in muscles because certain modulations of signaling pathways linked to insulin by different qualities of nitrogen intake may also have implications for cancer risks and development [43] and not only on the lifespan.

## 3. Essential Amino Acids and Cancer: What if the Substrates Provided by the Environment Change the Rules?

All mammals develop from a single cell, one oocyte fecundated by one spermatozoon. All cells in all tissues and organs have an origin from the rapid duplication of that cell, and all cells must withstand the same rules that allow development during foetal life: matter and energy should be constantly available in adequate amounts to promote the survival of any cell, thus maintaining the integrity of the organism. We have an incomplete understanding about both how memory and the mechanisms of this primordial time of our lives survive in cells, as well as how those are controlled by specific regulatory proteins, particularly in dividing cells [44]. Although protein synthesis suitable to match duplication needs is taken for granted, very few studies dealing with the dependence of the proliferation of cancer on essential amino acids have been published, and, among the few, only the role of specific amino acids has been studied.

As an example, recent and noteworthy, is a report on the metabolic dependence of embryonic stem cells on threonine, which is indispensable for biosynthesis but also for controlling signaling pathways that allow pluripotency and self-renewal. Embryonic stem cells have many features similar to cancer cells: for growth and proliferation; both require a catabolic transformation of nutrients into energy but also metabolic building blocks to meet the biosynthetic and anabolic needs. Additionally, embryonic stem cells use glycolysis to lactate as the preferred energy metabolic route to generate ATP, the metabolic adaptation known as “Warburg effect”. Accordingly, embryonic stem cells reportedly have less developed mitochondria and a lower oxygen consumption. Therefore, Chen and Wang [45] described the possible role in rapidly dividing cells of the threonine-dehydrogenase-mediated catabolism of threonine to glycine and acetyl-CoA, with the latter entering the citric acid cycle and promoting fatty acid synthesis through conversion to malonyl-CoA in mitochondria and export in the cytoplasm. In turn, glycine would contribute to one-carbon metabolism and methylation pathways, fuelling purine synthesis [46], as would be discussed in detail for serine/glycine metabolism in a specific paragraph.

Additionally, some aspects of the catabolism of tryptophan have been related specifically to cancer. Thus, depletion by indoleamine-2,3-dioxygenase (IDO) expressed either in tumour cells or antigen-presenting cells, as well as the production of an end product like kynurenine, has been related to immunosuppression in the cancer cellular microenvironment and in lymph nodes draining it, thus inducing T-cell anergy and apoptosis facilitating cancer expansion [47].

By contrast, follow-up studies of large populations have discovered that obese cirrhotic patients treated with high doses of branched-chain (BC) amino acids (AA), to prevent hepatic encephalopathy, had a reduced risk of liver cancer [48]. As possible mechanisms for those results, it was suggested that BCAA may act by inhibiting the insulin-induced PI3K/AKT signaling pathway through the active regulation of the serine/threonine kinase involved in the control of protein synthesis, cell growth, and metabolism, named the mammalian target of rapamycin (mTOR), targeting its functional complex mTORC1, which activates a feedback loop in PI3K signaling, and inhibiting the other complex mTORC2, thus controlling the genes related to apoptotic and anti-apoptotic pathways. The sum of those effects, finally, would foster cancer cell apoptosis [49].

Those observations are of interest because it is widely accepted that different essential amino acids, particularly leucine, but also isoleucine, methionine and, less potently, valine, control mTORC1 activation of protein synthesis [50].

## 4. Reducing the EAA/NEAA Ratios Drives Cancer Cells to Apoptosis, Activating Autophagy and Inhibiting the Proteasome

Indeed, we have shown that different types of cancer cells do not survive in a metabolic environment where EAA are largely prevalent in NEAA [20], a substrate-rich peculiar environment clearly beneficial for normal cells used as controls. A clear summary of the amino acids contained in the two formulations used in those experiments is reported in Table 1. Because some cancer cells were demonstrated to lose enzymes to synthesize specific non-essential amino acids (such as arginine, by cells lacking argininosuccinate synthase (AS-Sinth), which is indispensable for recycling citrulline to arginine) [51] while implementing the synthesis some NEAA, even if abundant in food and in circulating fluids, such as serine/glycine, the hypothesis was that cancer spares NEAA synthesis because it develops adapted to NEAA concentrations most abundant than EAA, as normally found in biological fluids. We suspected that cancer cells would not be suitable to manage specific alterations of EAA/NEAA ratios, as opposed to normal cells which maintain an expensive machinery to adapt to shortages of any NEAA and to the sudden availability of most scarcely available EAA. Indeed, we survive with limitations to our well-being even if undergoing caloric restriction, but we respond rapidly to EAA availability with increased synthesis even in chronic pathologies dominated by wasting [25]. Therefore, we showed that significantly reducing the EAA/NEAA ratio in the culture broth inhibits proteasome-linked proteolysis in cancer cells, while increasing autophagy, triggering an apoptotic drive [20], a response to certain stimuli that has already been analytically described and that is beclin-1 dependent [52].

Implications of these data are noticeable: for the first time, a certain fragility of cancer cells to modifications of metabolic environment is described that is, by contrast, favourable for normal cells, as documented in animals and humans and shown by previous findings [53,54,55] because the EAA/NEAA ratio triggers poly-adenosyl-di-phosphate ribose polymerase-1 (PARP-1) cleavage paralleled in various cancer cells by activating caspase 3 and driving the cells to undergo apoptosis. Additionally, an increased EAA/NEAA ratio modulates some activities of cathepsin B and L, and also increases p53 and p62SQSTM1, as well as beclin 1 and LC3BII, which exhibit autophagy activation. Additionally, EAA supplementation generated a marked inhibition of proteasome activity. Because EAA formulations were proven to be efficient in chronic heart failure clinical settings [56], while actual drugs promoting proteasome inhibition proved to be extremely toxic in cardiac cells, our findings open a new avenue of possibilities in supporting or enhancing chemotherapy effects, while protecting target organs, like the heart, from iatrogenic damage [57,58].

## 5. Serine and Glycine: Guilty of Feeding Cancer or Innocent Intermediates of Metabolism?

**Figure 1:**
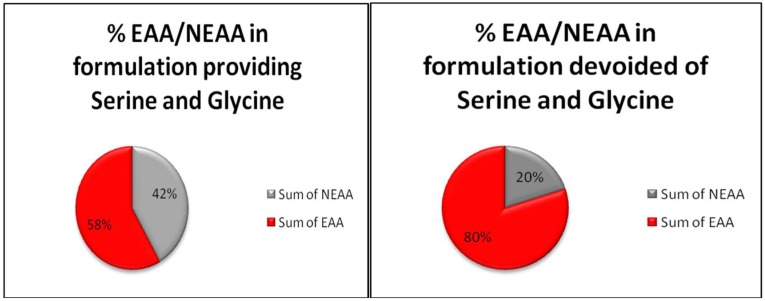
The figures are based on the methods used by Maddocks et al. [19]; citations 22 and 39. Composition of “Amino acid pre-mix”, providing both serine and glycine: arginine-HCl: 1.60%, l-cystine: 0.64%, l-glutamine: 1.60%, glycine: 1.33%, l-histidine-HCl: 0.80%, l-isoleucine: 1.07%, l-leucine: 1.60%, l-lysine-HCl: 1.87%, l-methionine: 0.80%, l-phenylalanine: 1.07%, l-serine: 1.33%, l-threonine: 1.07%, l-tryptophan: 0.27%, l-tyrosine: 0.53%, l-valine: 1.07%. Composition of “Amino acid premix” devoid of serine and glycine: l-arginine:-HCl: 1.60%, l-cystine: 0.64%, l-glutamine: 1.60%, l-histidine: 0.96%, l-isoleucine: 1.28%, l-leucine: 1.92%, l-lysine-HCl: 2.24%, l-methionine: 0.96%, l-phenylalanine: 1.28%, l-threonine: 1.28%, l-tryptophan: 0.32%, l-tyrosine: 0.64%, l-valine: 1.28%. The weight ratios among EAA (in red) versus NEAA (grey) provided by the two formulations according to the Methods described by Maddocks OD et al. Serine starvation induces stress and p53-dependent metabolic remodelling in cancer cells. Nature, 2013; 493, 542–546. It should be noticed that the percentages were calculated for exactly 16 g of the serine- and glycine-free formulation, and for 16.28 g, which is the sum of the weights of the amino acids declared in the formulation providing both serine and glycine. Ovalbumin, the reference protein for human nutrition, contains 44.4% EAA or an EAA/NEAA ratio of approximately 0.8. By contrast, in formulations used by Maddocks et al. (according to citation 19), if the content of amino acids would have been calculated in molar ratios (number of molecules), the EAA/NEAA molecular ratio would have been near 1.2 when serine and glycine were provided and near 2.9 when the formulation was modified by eliminating serine and glycine. In any case, the essential amino acid content of those formulations is quite far from the food content of amino acids.

A one-carbon metabolism necessary for nucleotides and NADPH production and methyl-group transfer from donors required to target modifications of DNA, RNA, and proteins all depend on the metabolism of serine. Serine is an interesting amino acid, commonly present in food proteins, but it is an easily synthesized gluconeogenic amino acid because its carbon skeleton is derived from 3-phospho-pyruvate originated by anaerobic glycolysis from glucose and then is transaminated on the α carbon by glutamine-3 phospho-pyruvate transaminase (PSAT1), and finally dephosphorylated by a phospho-serine dephosphorylase (PSPH) to serine. Some cancers depend on the enhanced activity of this synthetic pathway to maintain purine synthesis and, using specific inhibitors of endogenous serine synthesis, cell growth is retarded even in the presence of abundant exogenous serine [59]. Serine can also be derived from glycine, but this reaction consumes nicotine amide dinucleotide reduced form (NADH) and depletes the methyl group of folates by this metabolic pathway, providing modifications similar to those consequent to alcohol ingestion, a condition indeed also marked by homo-cysteine accumulation [60]. That ethanol consumption is linked to increased risk of cancer is well known [61], but the role of methyl group depletion of folates is more puzzling. Indeed, on the one hand, folate fortification of food has significantly reduced the incidence of colon cancer in the USA in a long-term epidemiological survey [62]; thus, repletion of folate reserves is favourable to prevent some cancers. On the other hand, a very high level of folate intake does not further improve protection [63]. Anti-folate molecules, developed on the clinical observations that dietary folate deficiency reduces the leukaemic cell number in acute leukaemia [64], remains one of the most efficient and widely used drug in the chemotherapy of different tumours [65]. Therefore, because the maintenance of folate efficiency is based on the continuous reloading of methyl groups at positions 5 and 10, and those methyl groups are necessarily derived from the serine to glycine metabolism through an energetically favourable reaction to produce NADH, certain roles of serine in cancer have been repeatedly suggested [66,67]. The transfer of methyl groups is fundamental for metabolism integrity, and some congenital alterations of the folate cycle increase cancer risk [68]. Although the different positions of methyl groups on the tetra-hydro-folate molecule signal their destinations, the dependency on serine to fully reload and reactivate folate efficiency is valid for both methyl groups at positions 5 and 10 on tetrahydrofolate (THF) molecules. Methylation is required for nucleotide synthesis, which is increased in cancer because it is related to rapid duplication. Thus, much work has focused on the role of serine/glycine in cancer cell metabolism based on the hypothesis that, by depriving serine/glycine cancer metabolism, the growth and development of cancer would be impaired. Because the depletion of methyl groups from THF is rapidly obtained by ethanol ingestion, and this relates to increased cancer risk in different tissues [69], there is some contradiction regarding how cancer occurs. Indeed, often researchers blame the utilization of single pathways indispensable for normal metabolism that are implemented although not structurally modified to fulfil the specific metabolic needs of cancer cells. From adenosine triphosphate (ATP) to membrane lipid synthesis, many, if not all, normal metabolic pathways must match an increased duplication need marking cancer cell aggression. In our opinion, it should not be forgotten that cancer cells originate from normal cells of normal tissues of normal organs and develop substantially bound to the same metabolic rules and activities of the cells from which they are derived but, evidently, with some metabolic advantage not yet evidenced that competitively select them [17]. Certain previous reports have focused on serine/glycine deprivation [19,70] and showed that cancer cells undergo metabolic derangement and lowered development when fed formulation deprived of serine and glycine. However, based on the data also presented in an interesting publication [27], the serine/glycine deprivation protocol is heavily biased by methodology and authors have fallen into a Stolzemberg’s trap [71] because they compared a formulation containing either EAA and NEAA, comprising serine and glycine among the NEAA, to the same formulation simply deprived of serine and glycine. By doing so, they made a methodological mistake because they did not maintain constant EAA/NEAA ratios by substituting serine and glycine with some other NEAA of their choice. Thus, they compared two different metabolic substrates and not principally for serine and glycine deprivation. Indeed, to appropriately test the hypothesis that serine and glycine are responsible for the maintenance of cancer cells and to test a formulation deprived of serine and glycine correctly, different from the protocol they used, they should have deprived the control formula of serine and glycine, but those amino acids should have been substituted with equimolar amounts of, as an example, glutamine, aspartate, glutamic acid or, at the least, arginine for serine, and alanine for glycine. By contrast, they tested not simply a formulation deprived of serine and glycine but a formulation providing an overwhelming EAA content with respect to NEAA, thus deeply altering the EAA/NEAA ratio, but still attributed findings to the simple substitution of serine and glycine, as shown in Figure 1.

Because we described [20] some of the mechanisms through which the increased EAA/NEAA ratios may be altered by the strict biological environment in which cancer cells metabolically proliferate, by altering the EAA/NEAA ratio, the fragility of cancer metabolism was unveiled to depend on the largest availability of NEAA with respect to EAA. These findings were observed by inverting the EAA/NEAA ratio usually present under physiological conditions (as in plasma and extracellular fluids) and constantly maintained both in human cells and in food proteins of any origin. Therefore, by supplementing two different formulations in which EAA were largely prevalent, we evidenced a significant activation of autophagy. This is probably linked to a reduced capacity of cancer cells to derive sufficient NEAA from the intermediates of glycolysis to match the huge needs of NEAA for duplication. Increased EAA in the cancer cell food based environment would imbalance nuclear signalling controls, as we showed at least at the level of poly ADP-ribose polymerase-1 (PARP-1), and this activity could trigger a cascade of events mortal for cancer cells because the elevated metabolic requirements of NEAA in cancer cells are devoted to matching both energy production and the synthesis of proteins. Therefore, an increased demand for NEAA, as a consequence, triggers autophagy. EAA in excess also blunt proteasome-dependent proteolysis, and this critical condition conflicting with increased autophagy finally triggers apoptosis. The substantial alteration of the EAA/NEAA ratio used in our study [20] is similar, even if EAA predominance was most strictly marked than that used by Maddocks et al. [19]. Therefore, because the alteration of the NEAA/EAA ratios was also induced by their protocol without full knowledge of the underlying cause, their most interesting results were misleadingly attributed only to the deprivation of serine and glycine. The methodological mistake was that the deprivation of serine and glycine from the amino acid formulation used in the control groups was done without fully compensating the weight or molar ratios with other NEAA so that they compared a totally unbalanced EAA/NEAA ratio where EAA molecules are prevalent. A schematic representation of the EAA and NEAA contents in the formulations used by Maddocks et al. is presented in Figure 1.

Notably, in our study, the presence of some serine, as well as some ornithine (an amino acid of metabolic interest that is not present in proteins but is a precursor or product of glutamine metabolism), linked to α-ketoglutarate (OKG, providing physiological precursors of glutamic acid and glutamine synthesis), potentiates the effects of the EAA-rich formulation and elicits specific epigenetic responses, one example of which is the different cathepsin B and L activation levels. EAA-rich formulations supplemented in the diet have already been shown to increase the lifespan by counteracting age-linked mitochondrial biogenesis losses [34], and different papers have shown, both in experimental and human settings, that lowering the EAA/NEAA ratios with some essential amino acids (the branched-chain ones) reduces liver damages and cancer risk [32,72]. Details of the underlying mechanisms of those behaviours are entangled and remain under study [43], although we believe that, by altering the EAA/NEAA ratios, a means of triggering a totally innovative network of relationships among key gatekeepers such as mTORC1 and 2 [73], AMPK and AKT [74], has been identified, unveiling some control of the proteolysis/autophagy network mastered by amino acid availability that is evidently related to apoptosis or perhaps to death due to the collapse of energy production, that is anoikis [75].

## 6. Glutamine, Proline, and Ornithine—Entangled Relationship

If serine is a key amino acid for the maintenance of methylation by one-carbon-linked biochemistry, glutamine is pivotal due to the relevance of its role as a nitrogen donor. Thus, glutamine is central for synthetic pathways driving to NEAA synthesis, although its carbon skeleton is extremely important also in supplying intermediates to the citric acid cycle, particularly providing α-ketoglutarate to the citric acid cycle and refueling a key oxaloacetate precursor of the mitochondrial acetate oxidation cycle (tricarboxylic or Kreb’s cycle).

Three different and excellent reviews have been published recently on the role of glutamine metabolism in cancer development and survival. All the reviews underscored how glutamine is necessary for purine and pyrimidine biosynthesis beyond being necessary for completing protein and also glucosamine synthesis in the extracellular matrix and it is required for cancer cell growth and duplication [7,76,77]. Glutamine is an amino acid synthesized easily in all cells containing transaminases. It is also present in erythrocytes. From the intermediate of glycolysis α-ketoglutarate (αKG), the first glutamate is synthesized by the transamination of one –NH_2_ group on carbon α and it is obtained by dismantling nitrogen from aspartate or alanine but also from essential amino acids such as leucine or valine. Next, glutamine is completed by glutamino-synthase, which transfers another amino group on carbon γ of glutamic acid. In turn, glutamine plays a central role in the synthesis of all NEAA because it provides nitrogen for the amination of different substrates derived from glycolytic metabolisms, such as pyruvate, oxaloacetate, and αKG. The pathway works in both ways. Thus, from glutamine, different key molecules connected with refuelling energy production, such as pyruvate, oxaloacetate, or αketo-glutarate, can be generated by transaminases that transfer –NH_2_, thus providing the maintenance of NEAA synthesis when and where necessary, or by transamidation that generates NH_3_/NH_4_. This metabolic pathway is important in kidneys where the excretion of NH_4_ allows for the control of K and Na reabsorption [78]. Glutamine, like the metabolically related amino acid arginine [79], under conditions of elevated metabolic requirements, may become “conditionally essential” because it is largely consumed to produce energy and may become insufficiently available for the maintenance of immune defence (lymphocytes and macrophages proliferation and metabolism) and absorption of nutrients to prevent malnutrition (enterocytes). Glutamine is also indispensable due to its role both as an energy substrate and as a precursor of nucleotide synthesis; thus, it is necessary for the maintenance of physiological rapid duplication for cells like enterocytes but also for leucocytes under life-threatening conditions, as in sepsis [80]. On the other hand, it has been clinically proven that EAA supplementation is highly effective in promoting immune system efficiency under those same conditions [81], and this may be an alternative to glutamine supplementation because EAA can promote glutamine endogenous synthesis locally but also protects against ammonia- and glutamine-dependent brain damage following excess ammonia production by enterocytes, in contrast to what happens with enterally provided glutamine [82]. Indeed, under normal conditions, enterocytes prevent glutamine absorption dismantling by the deamination of glutamine molecules by a specific phosphate-activated glutaminase prior to the absorption of glutamate, and so, producing ammonia that can be absorbed and generate the most severe neurological alterations in the case of liver impairment [83]. This chain of events unveiled the toxicity of the ammonia-dependent glutamine synthetic rate in neuronal/astrocyte metabolism and the protective role of some EAA, particularly leucine, that activate the export of glutamine from brain cells to treat hepatic encephalopathy [84]. Accordingly, the adverse effects of long-term glutamine supplementation have been largely discussed [85]. Briefly, they may be summarized by observing that exogenous glutamine suppresses the expression of glutamine-synthase [86]. Thus, patients treated with glutamine supplementation should be discontinued by carefully queuing therapy, or a sudden stop in supplementation would lead to endogenous synthesis by either glutamine starvation or the loss of immune efficiency [87].

In cancer cells, glutamine is consumed far more than predicted by the demands of protein synthesis [7], thus, increased energy costs may be suspected. Although various attempts to target glutamine metabolism in cancer-specific glutaminase isoforms and its variants have been explored, the understanding of the full picture of requirements of glutamine in tumour cells appears far from being completed. Additionally, metabolic heterogeneities linked to genetic instability and both the progression of mutations and complexity of the tumour metabolism and its relationship with cellular micro-environment and perfusion fluids indicate the need for more studies. This is in order to understand the regulation of tumour metabolism and whether the complex biochemical network, in which glutamine is at the centre of anaplerotic and cataplerotic metabolism, would be a suitable target for effective therapies [79].

Additionally, glutamine metabolism is extremely intertwined with the synthesis of many other different amino acids, such as ornithine and proline, whose origins are directly or indirectly connected to glutamine synthesis through glutamic acid. An important point of view is that which was proposed by Phang JM et al. [88], who underscored that, at the centre of glutamine, ornithine and proline metabolism, there is an intermediate common to all three amino acids, delta-pyrroline 5 carboxylic acid (P5C), that is critically dependent on proline and proline oxidase (POX, also known as PRODH, proline dehydrogenase) to be produced and to generate ATP in mitochondria. The recycling of P5C to proline by P5C reductase out of mitochondria where P5C is produced is indispensable to generate reduced NADP (NAD phosphate), thus fuelling the pentose phosphate cycle (PPP) and activating the synthesis of pentoses from glucose 6 phosphate with NADPH generation, which is useful for membrane lipid synthesis. When proline is available, POX/PODH increases the degradation of proline sequentially first to glutamate and then to αKG, and the POX/PODH-dependent production of αKG seems crucial in the downregulation of hypoxia-inducible factor-1 (HIF-1) signalling in cancer. Therefore, it is not surprising that, in many cancers, POX/PRODH is suppressed but is up-regulated by the tumour suppressor p53 [89].

Notably, αKG, more than being just an intermediate of the citric acid cycle by αKG-dehydrogenase to yield 6 ATPs and generate NADH, is the key molecule for the malate-αKG-aspartate shuttle and is an indispensable cosubstrate for transaminase to generate oxaloacetate, allowing aspartate to glutamate conversion and indirectly glutamine synthesis. Additionally, αKG plays multiple regulatory roles, from being an indispensable substrate for prolyl hydroxylases that mediate the ubiquitination and proteasomal degradation of hypoxia-inducible factor-1α (HIF-1α), and, if not oxidized to succinate, may increase DNA and histone hypermethylation [8]. In any case, cycling P5C produced from proline into mitochondria and reduction back to proline in the cytoplasm is considered a way to transfer reducing equivalents generated by the oxidation of glucose in the PPP into mitochondria to generate ATP [90]. Particularly interesting in cancer is the metabolic relationship between glutamine and proline, and, most significantly, what is most interesting may be both the synthesis of P5C from glutamate and metabolic interconversions of P5C to either proline or glutamate, or to ornithine first and then arginine. On the one hand, glutamate can provide αKG as a substrate for the citric acid cycle, thus generating a reactive oxygen species and signalling activation to autophagy and apoptosis. On the other side, glutamate, as P5C, may enter the proline cycle and recycle NADP to NADPH while fueling glucose into PPP [91]. NADPH is generated from PPP, which is necessary also to induce c-Myc oncogene-controlled transcription, which is dependent on adenosine sensing. Therefore, the nucleoside biosynthetic pathway, also dependent on P5C and glutamine, may connect c-Myc activation to tumour metabolism [92,93].

Because most ornithine is derived by the cleavage of urea from arginine, although not being a proteinogenic amino acid, ornithine is a key molecule to maintain nitrogen clearance through citrulline and back to arginine. The recycling of ornithine to arginine after urea cleavage is a pathway requiring both aspartate (derived from glutamine transamination) and *N*-acetyl-glutamate to form argininosuccinate. Interestingly, the key enzyme involved, arginino-succinate synthetase (AS-Sinth), directly regulates AMPK activation and lipid synthesis by consuming ATP to AMP, but ornithine is either the starting point for the synthesis of polyamines on the one hand but it is also a substrate to produce P5C and the strictly related glutamic-γ-semialdehyde (GSA). Thus, ornithine is also a precursor of proline synthesis. Notably, arginine may be produced in the liver and kidneys in both urea and polyamine through agmatine via an alternative route to the cycle producing ornithine and urea. Agmatine is a metabolite that is relatively poorly studied and is derived by arginine through arginine decarboxylase and is then converted by agmatinase to putrescine and urea, a minor pathway also identified in humans [94].

Additionally, some links between glutamine and serine metabolic pathways may be found in recent data showing that arginine deprivation in cells deficient in AS-Sinth (i.e., deficient in recycling ornithine to arginine after urea cleavage) either upregulates glutamine utilization into the citric acid cycle to increase mitochondrial activity or increases serine synthesis from pyruvate subtracting pyruvate to produce lactate by cytoplasmic glycolysis via stimulating phosphoglycerate dehydrogenase (PHGDH) and inhibiting pyruvate kinase isoform 2 (PKM2, particularly expressed by cancer cells) acting at either the total protein level and phosphorylation changes, a synergic activity finally resulting in a decreased Warburg phenotype [54]. However, whether the activation of PKM2 activity could blunt cancer development because it increases the mitochondrial oxidative phosphorylation to promote ROS production, is debated, although its deletion and inhibition of activity proved to worsen cancer evolution [95].

## 7. Conclusions

Complexity is elevated when dealing with metabolism and amino acids, and this is reflected by the various protocols used and different interpretations of findings when protocols are accurately analyzed. Recent data open new insights into cancer metabolism and a series of puzzling questions. Cancer develops and grows in the same metabolic environmental conditions (availability of substrates providing information and energy) of normal cells, but does cancer “eat” according to the same needs? Does it follow the same rules of normal cells to be competitive and proliferate?

In vitro studies have recently demonstrated that largely increasing the EAA/NEAA ratio or altering the reduced ratio (<<1) that is usually found in proteins and biological fluids blunts cancer development, and, although inadvertently demonstrated by some authors in vivo, supplies EAA in excess compared with NEAA obtained by subtracting serine and glycine from the growth medium as well as from pellets in studies in mice, apparently reducing cancer growth. EAA given in excess of NEAA to cancer cells modify different epigenetic and post-translational targets. EAA in excess of NEAA increase autophagy and PARP-1 cleavage, inhibit proteasome, and finally trigger apoptosis, while normal cells are unharmed by those modifications. In foods, as well as in human proteins and fluids, NEAA are more abundant than EAA, and supplementing large amounts of EAA could be a safe procedure suitable to slow duplication by metabolically stressing selectively cancer cells. EAA in excess of NEAA have unveiled a new specific fragility of cancer metabolism that warrants full explored for the possible future therapeutic solutions that this safe procedure may suggest.

## Figures and Tables

**Table 1:** The AAs ratios in the formulations tested by Bonfili et al. [20], expressed as percentages of 100 g.

**100% EAAs (*w*/*w* %)**							
Leucine	Isoleucine	Valine	Histidine	Lysine	Threonine	Methionine *	Phenylalanine
31.25	15.625	15.625	3.75	16.25	8.75	1.25	2.5
Tryptophan	Tyrosine **	Cystine *					
0.5	0.75	3.75					
**85% EAAs and 15% NEAAs (*w*/*w* %)**							
Leucine	Isoleucine	Valine	Histidine	Lysine	Threonine	Methionine *	Phenylalanine
13.53	9.65	9.65	11.60	11.60	8.70	4.35	7.73
Tryptophan	Tyrosine **	Cystine *	Serine	N-Acetyl Cysteine *		Ornithine-α Ketoglutarate	
3.38	5.80	8.20	2.42	0.97		2.42	

* Both formulations contain cystine (and also *N*-acetylated cysteine in the 85% NEAA and 15% EAA formulations,) summed to methionine to match sulphur-containing AA needs minimizing the possible homo-cyst(e)ine toxicity by 20. ** Tyrosine is present in both formulations because, when calculating phenylalanine needs, it was considered that tyrosine is an NEAA only for the liver and partially for the kidneys, which can derive it by hydroxylating phenylalanine, whereas it is fully essential for any other cell of the body.
